# Will the untreated ulnar styloid fracture influence the outcome of unstable distal radial fracture treated with external fixation when the distal radioulnar joint is stable

**DOI:** 10.1186/1471-2474-14-186

**Published:** 2013-06-12

**Authors:** Yi-xin Chen, Xin Zheng, Hong-fei Shi, Yu-fan Wangyang, Han Yuan, Xiao-xiao Xie, Dong-ya Li, Chang-jun Wang, Xu-sheng Qiu

**Affiliations:** 1 Department of Orthopaedics, Nanjing Drum Tower Hospital, The Affiliated Hospital of Nanjing University Medical School, No. 321 Zhongshan Road, Nanjing, China

**Keywords:** Distal radius fracture, Ulnar styloid fracture, External fixation

## Abstract

**Background:**

The ulnar styloid is an important supportive structure for the triangular fibrocartilage complex. However, it remains inconclusive whether or not a fractured ulnar styloid should be fixed in an unstable distal radius fracture (DRF) with a stable distal radioulnar joint (DRUJ). The purpose of this study is to evaluate the effect of an untreated ulnar styloid fracture on the outcome of unstable DRF treated with transarticular external fixation when the DRUJ is stable.

**Methods:**

106 patients with an unstable DRF and a stable DRUJ were included in this study following external fixation. The patients were divided into the non-fracture, the tip-fracture and the base-fracture groups according to the location of the ulnar styloid fracture at the time of injury. Postoperative evaluation included the range of wrist motion, the radiological index, the grip strength, the PRWE-HK scores, the wrist pain scores, and the instability of DRUJ at the external fixator removal time, three months postoperatively and the final follow-up visit.

**Results:**

The patients were followed for 12 to 24 months (15 months in average). Sixty-two of 106 patients (58%) had ulnar styloid fracture and 16 patients (26%) showed radiographic evidence of union of ulnar styloid fractures at the final follow-up visit. No significant difference in the radiological findings, the range of wrist motion, the grip strength, the PRWE-HK scores, and the wrist pain scores among three patient groups was detected at the external fixator removal time, three months postoperatively, or the final follow-up visit. Six of the 106 patients (5.7%) complained of persistent ulnar-side wrist pain during daily activities. One patient (0.9%) showed a positive sign in a stress-test, three patients (2.8%) showed a positive sign in a provocative-test, and five patients (4.7%) showed a positive sign in a press-test. There was no significant difference in the percentages of patients who complained of persistent ulnar-side wrist pain or showed a positive sign in the physical examination of the distal radioulnar joint among the three groups at the final follow-up time points.

**Conclusion:**

When the DRUJ is stable, an untreated ulnar styloid fracture does not affect the wrist outcome of the patient with an unstable DRF treated with external fixation.

## Background

The unstable distal radius fracture (DRF) is a common injury that requires operative treatment [1,2]. A wide range of fixation choices, such as locking-plate, external fixator, Kirschner-wire (K-wire), or the combination of these methods, are available for the unstable DRF [3,4]. With each fixation method showing advantages and disadvantage, there is still no sufficient clinical evidence to recommend one form of treatment over the others [4,5]. In prospective randomized trials comparing the method of external fixation combined with percutaneous pinning and open reduction and locking-plate fixation, excellent outcomes were observed in both methods at one year follow-up [6,7], with minimal differences in strength, motion, and radiographic alignment. More recently, Wei et al. [8] found that open reduction with internal fixation yields significantly better functional outcomes, forearm supination and restoration of anatomic volar tilt in a systematic review and meta-analysis of comparative clinical studies on the unstable DRF. However, external fixation led to better grip strength and wrist flexion. Given that external fixation is less invasive compared with internal fixation, it is generally agreed that external fixation plays an important role in treating the unstable DRF [2,6,7,9-12].

The unstable DRF is frequently associated with an ulnar styloid fracture, whose effect on the outcome of the treatment of the unstable DRF is still unclear, with the literature reporting inconclusive findings. Anatomical and biomechanical studies have determined that the ulnar styloid is an important supportive structure for the triangular fibrocartilage complex (TFCC) [13]. Some authors asserted that untreated ulnar styloid fracture is associated with damaged wrist functional outcomes and distal radioulnar joint instability [14-17]. In a recent cohort study involving 320 patients with DRFs treated operatively or non-operatively, Chan et al. [18] found that DRF associated with a base fracture of the ulnar styloid resulted in a higher rate of patient reported pain and disability when compared to isolated DRF. By contrast, Kim et al. [19], Sammer et al. [20] and Souer et al. [21] reported that the size of the ulnar styloid fracture, the degree of displacement and the healing status of the ulnar styloid did not affect the wrist function if the DRF was treated by plate fixation.

To our knowledge, previous research mainly focused on the influence of ulnar styloid fracture on the outcome of DRF treated with plate fixation [19-21]. Only one study reported the effect of ulnar styloid fracture on the outcome of DRF treated with transarticular external fixation or Kirschner-wire fixation [22]. In their study, Belloti et al. [22] found that patients with a DRF associated with an ulnar styloid fracture had worse scores in Disability of the Arm, Shoulder, and Hand (DASH) questionnaire; they suggested that an ulnar styloid fracture may be a predictive factor of poor wrist function if DRF was treated by external fixation or Kirschner-wire fixation. In our clinical practice, most of the unstable DRF patients were treated with closed reduction and external fixation augmented with percutaneous Kirschner-wires. The purpose of our study was to evaluate the effect of an untreated ulnar styloid fracture on the outcome of treating DRF with transarticular external fixation.

## Methods

### Patient inclusion criteria

Between January 2009 and March 2011, 106 patients with unstable DRF and stable DRUJ were involved in this study (Table 1). DRF was considered unstable according to the radiographic criteria described in literature [1,2,23]: (1) the initial dorsal angulation >20°, (2) with dorsal or volar comminution of the metaphysis, (3) shortening of radius >5 mm, or (4) with an associated ulnar fracture. Additionally, fractures were also considered unstable if radiographic displacements occurred after primary closed reduction and splinting [24]: shortening of radius >2 mm, joint fragment displacement >2 mm, or dorsal angulation >10°. The stability of DRUJ was checked during the surgical management.

**Table 1:** Patient demographics

	**Non-fracture**	**Tip-fracture**	**Base-fracture**
Gender (No. of patients)			
Male	20	12	21
Female	24	8	21
Total	44	20	42
Mean ages (years)	50.7±10.3	51.0±11.9	51.7±13.5

The patients were assigned into three groups according to the fracture characteristics of the ulnar styloid measured on anterior-posterior digital radiographs [25]: 44 patients had no fracture of ulnar styloid (non-fracture group), 20 patients had a fracture of the tip of ulnar styloid (tip-fracture group), and 42 patients had a fracture of the base of ulnar styloid (base-fracture group). The protocols and the procedure were approved by the Committee on Medical Ethics of Nanjing Drum Tower Hospital. Written informed consents were obtained from all of the participants in this study.

### Surgical technique

**Figure 1:**
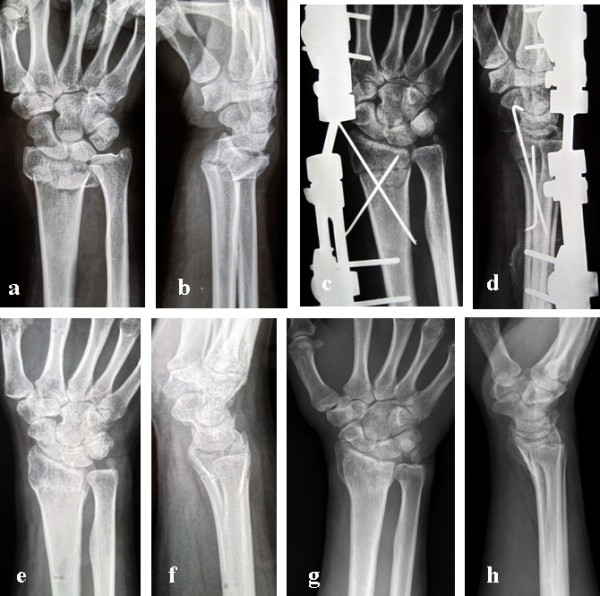
**The 65-year-old male patient with unstable distal radius fracture.** (**a**, **b**) The initial anterior-posterior and lateral radiographs of the distal radius showed an unstable fracture accompanied with the ulnar styloid base fracture: dorsal angulation at 30°; shortening of radius 7 mm. (**c**, **d**) The radiographs at the external fixator removal time: the fracture was treated with external fixation augmented with percutaneous Kirschner wires. (**e**, **f**) The radiographs at three months postoperatively: the distal radius fracture had united but the ulnar styloid base fracture had not. (**g**, **h**) The radiographs at the final follow-up visit: the ulnas styloid base fracture still had not united. Written informed consent was obtained from the patient to show the information here.

With general or brachial plexus anesthesia, distal radius fractures were reduced and fixed with external fixator and K-wires according to the standard technique described in literature [6]. In brief, threaded half-pins (2.5 mm in diameter) were inserted in the dorsoradial aspect of the metacarpal shaft of the index finger as well as the radial shaft 5 to 10 cm proximal to the fracture site through small incisions [6]. Under fluoroscopic guidance, the DRF was reduced closely and fixed with percutaneous K-wires (1.2 or 1.5 mm in diameter). Then the bridging external fixator was applied to stabilize the fracture (Figure 1). If a satisfactory reduction of the DRF or a smooth interface between the ulnar head and the sigmoid notch could not be obtained via close reduction, limited open reduction was performed [24]. In this series, totally 29 patients underwent limited open reduction.

After fixation of the DRF, the stability of the distal radioulnar joint (DRUJ) was checked [19]. Only the patients with a stable DRUJ were included in this study, and the distal ulnar styloid fracture was left untreated despite of the size or displacement of the fragment. The patients with DRUJ instability who required ulnar styloid fracture fixation were excluded from this study. After surgery, all patients underwent hand therapy including edema control, active and passive finger motion and forearm rotation. The K-wires and external fixator were removed 6–8 weeks after surgery.

### Clinical evaluation

Radiological and clinical assessments were performed at three time point: the external fixator removal, three months postoperatively and the final follow-up visit. Standard anterior-posterior and lateral radiographs were obtained at each time point to evaluate fracture healing and the alignment of the distal part of the radius. Radial inclination, volar tilt, and radial height were measured with the techniques described by Kereder et al. [26]. Wrist motion ranges, in terms of the extension, flexion, radial deviation, ulnar deviation, forearm pronation and forearm supination, were measured. The patients were also asked to complete the Patient-rated Wrist Evaluation Form (Hong Kong Version: PRWE-HK) [27], and rate their wrist pain both at rest and in motion on a 10-point visual analogue scale at each follow-up time point. The grip strength was evaluated by JAMAR hand dynamometer (Homecraft Ltd.)

The presence of ulnar-sided wrist pain during daily activities was recorded. Provocative-test, stress-test and press-test were performed on both the injured and the uninjured sides at the final follow-up time point to evaluate the characteristics of the DRUJ. The provocative-test was performed to detect ulnar styloid impaction syndrome [28]: the examiner positioned the forearm in neutral rotation, and then maximally extended the wrist and rolled the forearm into maximum supination; if this maneuver produces localized pain at the ulnar styloid, findings are considered positive. The stress-test was used to detect the DRUJ stability [29]: the distal radius was stabilized by the examiner, and then the distal ulna was translated dorsally and volarly with the forearm in neutral position, pronation, and supination; if the greater laxity and pain of the DRUJ was present, findings were considered positive. The press-test creates an axial ulnar load and has high sensitivity for detecting a tear of the triangular fibrocartilage complex [30]: the patient was asked to grip both sides of a chair and pushed himself or herself up from a seated position; a positive press-test causes focal ulnar-sided wrist pain.

### Statistical analysis

The statistical differences of the age, radiological finding, range of motion and functional outcomes were detected by means of one-way ANOVA (S-N-K) if the variances were homogeneous; but if not, Kruskal-Wallis test was used. Chi-square test was used to evaluate the differences of the gender and the union rate of the fracture of ulnar styloid. Fisher’s exact test was used to detect the differences of the positive rate of the wrist physical examination. The SPSS version 15.0 software (SPSS Inc, Chicago, IL, USA) was used and statistical significance was accepted at P < 0.05.

## Results

### Fracture union rate

All of the patients were followed for 12 to 24 months (15 months in average). All 106 unstable DRFs had united by three months postoperatively. 62 of 106 patients (58%) had ulnar styloid fracture and 16 patients (26%) showed radiographic evidence of the union of ulnar styloid fractures at the final follow-up visit. The union rate of the ulnar styloid fracture was 30% (6 of 20 patients) in the tip-fracture group, and 24% (10 of 42 patients) in the base-fracture group with no significant difference detected between the two groups (p=0.603). No significant difference was observed considering age (p=0.684) and gender (p=0.559) among the three patient groups (the no-fracture group, the tip-fracture group, and the base-fracture group) (Table 1).

### Radiological findings

There was no significant difference in the radial inclination angle, the volar tilt angle and the radial height among the three patient groups at the three time points (at external fixator removal, at three months postoperatively, and at final follow-up visit). Compared with the radiological findings at the external fixator removal time, no significant differences were observed at three months postoperatively and at the final follow-up visit (Table 2).

**Table 2:** Radiological findings

	**At external fixator removal**	**At 3 months**	**At final follow-up**
Radial inclination (deg.)			
Non-fracture	22.5 ± 3.5	21.2 ± 3.3	21.3 ± 2.7
Tip-fracture	20.8 ± 4.3	20.3 ± 4.3	20.6 ± 3.9
Base-fracture	21.9 ±3.4	21.4 ± 3.3	21.5 ± 3.1
P value	0.210	0.496	0.617
Volar tilt (deg.)			
Non-fracture	3.0 ± 1.8	2.8 ± 1.5	2.9 ± 1.2
Tip-fracture	2.7 ± 2.2	2.5 ± 1.3	2.6± 1.0
Base-fracture	3.4 ± 2.2	3.2 ± 2.2	3.4± 2.2
P value	0.471	0.404	0.139
Radial height (mm)			
Non-fracture	11.4 ± 3.1	11.3 ± 2.6	11.3 ± 2.6
Tip-fracture	13.0 ±3.1	12.5 ± 3.0	11.8 ± 2.9
Base-fracture	12.0 ± 2.9	11.6 ± 2.6	11.7 ± 2.5
P value	0.471	0.261	0.681

Data are expressed as means ±SD.

### Range of wrist motion

There were no significant differences in the range of wrist motion among the three patients groups at the three time points. However, compared with the range of wrist motion at the external fixator removal time, the wrist extension, wrist flexion, radial deviation, ulnar deviation, forearm pronation and forearm supination were significantly improved at three months postoperatively and at the final follow-up visit (Table 3).

**Table 3:** Range of motion

	**At external fixator removal**	**At 3 months**	**At final follow-up**
Wrist Extension (deg.)			
Non-fracture	25 ± 9	45 ± 12^﹡﹡^	51 ± 8^﹡﹡﹟^
Tip-fracture	28 ± 9	48± 10^﹡﹡^	53 ± 10^﹡﹡﹟^
Base-fracture	23 ± 8	46 ± 12^﹡﹡^	52 ± 9^﹡﹡﹟^
P value	0.182	0.456	0.721
Wrist Flexion (deg.)			
Non-fracture	34 ± 10	49 ± 7^﹡﹡^	57 ± 9^﹡﹡﹟﹟^
Tip-fracture	37 ± 10	46 ± 9^﹡﹡^	56 ± 8^﹡﹡﹟^
Base-fracture	38 ± 9	47± 7^﹡﹡^	58 ± 10^﹡﹡﹟^
P value	0.172	0.481	0.558
Radial Deviation (deg.)			
Non-fracture	10 ± 3	18 ± 5^﹡﹡^	22 ± 6^﹡﹡﹟﹟^
Tip-fracture	11 ± 3	17 ± 5^﹡﹡^	23 ± 6^﹡﹡﹟﹟^
Base-fracture	11 ± 4	16 ± 5^﹡﹡^	22 ± 6^﹡﹡﹟﹟^
P value	0.496	0.392	0.624
Ulnar Deviation (deg.)			
Non-fracture	22 ± 7	28 ± 11^﹡﹡^	35 ± 8^﹡﹡﹟﹟^
Tip-fracture	20 ± 9	26 ± 10^﹡^	39 ± 11^﹡﹡﹟﹟^
Base-fracture	21 ± 7	27 ± 9^﹡^	36 ± 11^﹡﹡﹟﹟^
P value	0.382	0.340	0.331
Forearm Pronation (deg.)			
Non-fracture	61 ± 12	78 ± 11^﹡﹡^	81 ± 12^﹡﹡^
Tip-fracture	60 ± 11	78 ± 10^﹡﹡^	81 ± 9^﹡﹡^
Base-fracture	64 ± 10	81 ± 7^﹡﹡^	83 ± 9^﹡﹡^
P value	0.434	0.516	0.780
Forearm Supination (deg.)			
Non-fracture	62 ± 13	78 ± 13^﹡﹡^	82 ± 14^﹡﹡^
Tip-fracture	66 ± 10	80 ± 9^﹡﹡^	81 ± 10^﹡﹡^
Base-fracture	64 ± 11	78 ± 12^﹡﹡^	83 ± 13^﹡﹡^
P value	0.430	0.512	0.583

Data are expressed as means ±SD. Compared with the data at the external fixator removal time: p<0.05^﹡^, p<0.01^﹡﹡^. Compared with the data at three months postoperatively: p<0.05^﹟^, p<0.01^﹟﹟^.

### Functional outcomes

There was no significant difference in the scores of PRWE-HK, the scores of pain in motion, the scores of pain at rest and the value of the grip strength among the three patient groups at three time points. However, compared with the wrist function at the external fixator removal time, the wrist functions were significantly improved at three months postoperatively and at the final follow-up visit (Table 4).

**Table 4:** Functional outcomes

	**At external fixator removal**	**At 3 months**	**At final follow-up**
PRWE-HK scores (points)			
Non-fracture	59.7 ± 18.3	42.6 ± 27.9^﹡﹡^	16.1 ±12.5^﹡﹡﹟﹟^
Tip-fracture	58.1 ±21.3	38.7 ±21.0^﹡﹡^	18.5 ±13.0^﹡﹡﹟﹟^
Base-fracture	56.2 ± 18.9	46.7 ± 22.6^﹡﹡^	14.5 ± 9.9^﹡﹡﹟﹟^
P value	0.694	0.476	0.452
Pain in motion(1–10 points)			
Non-fracture	6.0 ± 1.6	4.4 ± 2.0^﹡﹡^	1.7 ± 1.8^﹡﹡﹟﹟^
Tip-fracture	6.1 ± 1.9	4.3 ± 1.6^﹡﹡^	1.3 ± 1.6^﹡﹡﹟﹟^
Base-fracture	5.9 ± 1.5	3.9 ± 1.7^﹡﹡^	1.4 ± 1.5^﹡﹡﹟﹟^
P value	0.894	0.450	0.541
Pain at rest(1–10 points)			
Non-fracture	1.9 ± 1.2	1.2 ± 1.1^﹡^	0.9 ± 1.4^﹡﹡^
Tip-fracture	1.8 ± 1.1	1.1 ± 0.8^﹡^	0.8± 1.0^﹡﹡^
Base-fracture	1.9 ± 1.6	0.9 ± 1.1^﹡﹡^	0.5±0.9^﹡﹡^
P value	0.947	0.369	0.555
Grip strength (kg)			
Non-fracture	13.8 ± 4.5	20.2 ± 6.5^﹡﹡^	25.2 ± 7.1^﹡﹡﹟﹟^
Tip-fracture	14.6 ± 5.1	18.5 ± 4.8^﹡^	25.0 ± 5.3^﹡﹡﹟﹟^
Base-fracture	14.4 ± 5.6	19.7± 5.4^﹡﹡^	26.6 ± 8.5^﹡﹡﹟﹟^
P value	0.783	0.542	0.576

Data are expressed as means ±SD. Compared with the data at the external fixator removal time: p<0.05^﹡^, p<0.01^﹡﹡^. Compared with the data at three months postoperatively: p<0.05^﹟^, p<0.01^﹟﹟^.

### Clinical characteristics of the DRUJ at the final follow-up visit

During the data collection period, six of the 106 patients (5.7%) complained of persistent ulnar-side wrist pain during daily activities. One patient (0.9%) showed a positive sign in a stress-test, three patients (2.8%) showed a positive sign in a provocative-test, and five patients (4.7%) showed a positive sign in a press-test. There was no significant difference among the three groups in the percentages of patients who complained of persistent ulnar-side wrist pain or showed a positive sign in the physical examination of DRUJ at the final follow-up visit (Table 5).

**Table 5:** Clinical characteristics of the DRUJ at the final follow-up

	**Non-fracture (%)**	**Tip-fracture (%)**	**Base-fracture (%)**	**P value**
Ulnar-side pain	6.8 (3/44)	5 (1/20)	4.8 (2/42)	0.909
Positive Stress-test	2.3 (1/44)	0 (0/20)	0 (0/42)	1.000
Positive Provocative-test	4.5 (2/44)	5 (1/20)	0 (0/42)	0.406
Positive Press-test	4.5 (2/44)	5 (1/20)	4.8 (2/42)	0.997

### Complications

**Figure 2:**
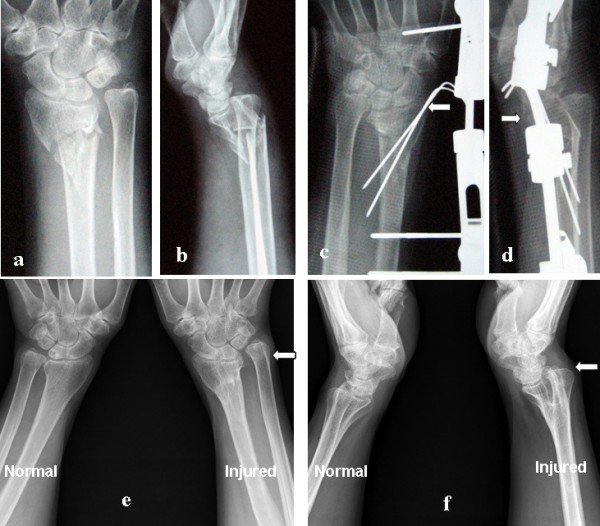
**The 56-year-old female patient with unstable distal radius fracture.** (**a**, **b**) The initial anterior-posterior and lateral radiographs of the distal radius showed an unstable fracture without an ulnar styloid fracture: volar comminution of the metaphysis. (**c**, **d**) Three weeks postoperatively, the fracture segments were displaced (white arrow); the patient declined re-operation and her wrist was fixed with above-the-elbow plaster. (**e**, **f**) Two years postoperatively, the distal radius fracture had mal-united with radius shortening and instability of the distal radioulnar joint (white arrow). Written informed consent was obtained from the patient to show the information here.

Eight patients had a superficial pin-track infection. All infections were resolved after local wound cleaning and treatment with antibiotics. One patient had numbness in the first finger web without apparently improvement at the final follow-up visit. One patient showed the malunion of the DRF (Figure 2).

## Discussion

Ulnar styloid fractures are commonly associated with DRF. The rate of ulnar styloid fracture in our study was 58%. Our results were consistent with previous reports that ulnar styloid fractures are usually present in 50% to 65% of the DRF [17,19,21,25,31]. The incidence of the TFCC tear complicated to the DRF was reported to vary widely from 9% to 84% [32-36]. Lindau et al. [32] reported that 43 of the 51 patients (84%) were detected by arthroscopy to have complete or partial TFCC tears at the time of fracture. Richards et al. [33] showed that 35% of intra-articular fractures and 53% of extra-articular fractures in 118 patients had TFCC tears identified by arthroscopy. In contrast, Spence et al. [34] found that only two of 21 patients (9%) with the DRFs were identified by wrist MRI to have TFCC tear. He suggested that the TFCC tear was infrequently associated with the DRF and the incidence of TFCC tear might be overestimated by arthroscopy. He explained that since most DRFs occur in the elderly; age-related degenerative changes may contribute to the high rate of the TFCC tear detected by arthroscopy. Spence et al.’s suggestion was supported by the study of Wright et al. [37], who examined 62 cadaver wrists to determine the incidence of pathologic changes in asymptomatic elderly wrists, and found that TFCC tear was present in 53% of the cadaver wrists. In the present study, we performed a press-test at the final follow-up visit, which creates an axial ulnar load and has high sensitivity for detecting a tear of TFCC [30], to detect the incidence of the TFCC tear. We found that only five of 106 patents (4.7%) showed a positive sign. Our incidence of the TFCC tear was similarly low to that found by Spence et al., and our results indicated that the symptomatic TFCC tear may be infrequently complicated to the elderly with unstable DRF.

Concerns about DRUJ instability in ulnar styloid fractures derive from the fact that the TFCC inserts to the base of the ulnar styloid and plays the essential soft-tissue stabilizer of the DRUJ [29,38]. Until now, the relationship between ulnar styloid fractures and TFCC tear is still not established. Richards et al. [33] reported that no statistical correlation between ulnar styloid fractures and TFCC injuries could be found in the elderly with a mean age of 54 years. Similar to Richards et al.’s finding, our study showed that the incidence of the symptomatic TFCC tear in the patients with a mean age of 51 years detected by the press-test was 4.5% in the non-fracture group, 5% in the tip-fracture group and 4.8% in the base-fracture group. There was no significant difference (p=0.997) in the incidence of the TFCC tear among the three groups in our study.

May et al. [14] reviewed retrospectively 166 patients with DRFs and found that all DRFs complicated by DRUJ instability were accompanied by an ulnar styloid fracture. However, Lindau et al. [32] tried to detect the relationship between the ulnar styloid fracture and the DRUJ instability, and found that DRUJ instability was not correlated to the initial ulnar styloid fracture (p=0.53) or the ulnar styloid nonunion (p=0.32). During our data collection, only three patients were found intraoperatively to have DRUJ instability, and the occurrence of DRUJ instability in the patients with unstable DRFs was only 2.8%. Our results indicated that the unstable DRF might be infrequently complicated with DRUJ instability. These findings were also in line with some previous studies [20,35,36]. In particular, Sammer et al. [20] reported that the incidence of DRUJ instability complicated to DRF was only 2%; while Lindau et al. [36] reported that no DRUJ instability was found in 50 young adults with intra-articular DRFs.

Anatomic and biomechanical studies have demonstrated that, besides the TFCC, the distal interosseous membrane, the extensor carpi ulnaris, the pronator quadratus and the congruence between the sigmoid notch of the distal radius and the ulnar head all contribute to the DRUJ stability [29,38]. These studies explained why DRUJ instability complicated to DRF rarely occurred. Clearly, some ulnar styloid fractures do result in DRUJ instability, and we believe that treating the ulnar styloid fracture with open reduction and internal fixation is generally supported. The question we attempted to answer in the present study was if the DRUJ is stable, what effect an ulnar styloid fracture may have on the outcome of the DRF. In our study of the patients whose unstable DRF had been treated with closed reduction and external fixation, we were unable to detect any significant difference in the radiological findings, the range of wrist motion, the grip strength, the PRWE-HK scores, and the wrist pain scores among the three patient groups at the external fixator removal time, three months postoperatively and the final follow-up visit. Our results suggest that an untreated ulnar styloid fracture does not affect the wrist outcomes of the patient with unstable DRF, provided that the patient’s DRUJ is stable.

Ulnar-sided wrist pains were sometimes complained by the patients with unstable DRF. In our study, six of the 106 patients (5.7%) complained of persistent ulnar-sided wrist pain during daily activities at the final follow-up visit, with no significant difference in the incidence of the ulnar-sided wrist pain obtained among the three patient groups. Our findings were in agreement with the study reported by Zenke et al. [25], where the incidence of the ulnar-sided wrist pain complicated to DRF treated by volar plating was 16% at three months, 8.5% at six months, and 5.1% at twelve months postoperatively. In our study, among the six patients mentioned above, two showed distal radius shortening and a positive sign in the provocative-test; one showed malunion of the DRF and positive signs in the stress-test, provocative-test and press-test (Figure 2); and one presented crepitus during forearm rotation. Our results indicated that ulnar-sided wrist pain can be caused by ulnar styloid impaction, distal radius shortening, malunion of the distal radius, incongruity of the distal radio-ulnar joint, the injury of TFCC, or a combination of these causes.

Two limitations can be noted of our research. We relied mainly on the press-test and the stress-test to evaluate the incidence of TFCC tear and DRUJ instability. The two detecting methods are somewhat subjective, by being dependent on the researchers’ judgment. Another weakness is that there was no control group in which the ulnar styloid fracture was surgically treated. However, we believe convincing evidence has been presented in this paper to demonstrate that an untreated ulnar styloid fracture does not affect the wrist outcomes of a patient with unstable DRF treated with external fixation, if the patient’s DRUJ is stable.

## Competing interests

The authors declare they have no competing interests.

## Authors’ contributions

YXC and HFS participated in study design and manuscript drafting. YFW and XZ carried out the clinical outcome analysis. HY, XXX, DYL, and CJW performed data collection and radiological analysis. XSQ assisted in statistical analysis. All authors read and approved the final manuscript.

## Pre-publication history

The pre-publication history for this paper can be accessed here:

http://www.biomedcentral.com/1471-2474/14/186/prepub
